# What Is Safe and How Much Does It Matter? Food Vendors’ and Consumers’ Views on Food Safety in Urban Nigeria

**DOI:** 10.3390/foods11020225

**Published:** 2022-01-14

**Authors:** Stella Nordhagen, James Lee, Nwando Onuigbo-Chatta, Augustine Okoruwa, Eva Monterrosa, Elisabetta Lambertini, Gretel H. Pelto

**Affiliations:** 1 Global Alliance for Improved Nutrition (GAIN), 1202 Geneva, Switzerland; emonterrosa@gainhealth.org; 2 Independent Researcher, Toronto, ON M6G 2K4, Canada; leejam17@yahoo.com; 3 Global Alliance for Improved Nutrition (GAIN), Abuja 900103, Nigeria; nonuigbo-chatta@gainhealth.org (N.O.-C.); aokoruwa@gainhealth.org (A.O.); 4 Global Alliance for Improved Nutrition (GAIN), Washington, DC 20036, USA; elambertini@gainhealth.org; 5 College of Human Ecology, Cornell University, Ithaca, NY 14850, USA; gp32@cornell.edu

**Keywords:** foodborne illness, food choice, ethnographic research, traditional markets, sociocultural beliefs and practices, food safety attitude, knowledge and behavior

## Abstract

This study examines the food safety beliefs of vendors and consumers in a mid-sized Nigerian city using data from in-depth interviews and cognitive mapping techniques drawn from ethnography. We examine vendors’ and consumers’ perspectives on which foods are safe, which are not, and why; the place of foodborne illness among other health concerns and motivators of food choice; and how salient food safety is as a concern for vendors. The main perceived causes of unsafe food were found to be chemicals and insects; while bacterial illnesses were widely mentioned as a cause of gastrointestinal symptoms, these were not necessarily linked to food in consumers’ minds. Respondents agreed strongly that certain foods (e.g., cowpea, beef, green leafy vegetables, and local rice) were less safe than others. The importance of food safety as a choice motivator among consumers varies depending on framing: when asked directly, it was prominent and closely related to visible cleanliness, but concerns about food safety competed in consumers’ minds against other salient motivators of food and vendor choice, such as price. Most vendors did not see food safety, cleanliness, or hygiene as a key trait of a successful vendor, and just over half of vendors had any concern about the safety of their food. In conclusion, we note the implications for intervention designs, particularly the need to build upon consumers’ and vendors’ current beliefs and practices related to food safety in order to make foodborne disease prevention a more salient concern in food choice.

## 1. Introduction

Food safety, the assurance that food will not cause harm to the consumer when it is prepared or eaten according to its intended use [1], is necessary for food security and improving health, nutrition, and wellbeing [2]. For food to be safe, it should not contain harmful levels of hazards such as viruses, bacteria, molds, protozoa, helminths (worms), as well as chemicals associated with adverse health impacts [3]. Currently, foodborne diseases associated with 31 high-risk microbial and chemical hazards are responsible for an estimated 600 million illnesses and 420,000 premature deaths annually [3], mostly among those living in low- and middle-income countries (LMICs) [4,5] who make up about 75% of deaths (but only 41% of the global population). The per-capita burden is largest in Africa, at about 27 times the level seen in Europe or North America [3]. Foodborne illness also comes with a large economic price, estimated at about 20 billion USD per year, due to sickness and loss of life, treatment costs, and impacts on trade [2].

Transitioning lower-middle-income countries are the settings in which food safety generally reaches a critical point due to rapid economic, demographic, and dietary change amid limited (government and private-sector) capacity to manage food safety [2]. A prime example of this is Nigeria. Past studies have shown high levels (contamination in up to 100% of samples) of risk and hazard for numerous foods sold in the country’s traditional markets [6], and the WHO region containing Nigeria has the highest per capita burden of foodborne illness in the world [3].

There is thus a need to improve food safety in Nigeria. Doing so requires understanding, and potentially shaping, the motivations and beliefs that impact the decisions of consumers and food vendors, in order to enable consumers to demand safer food and vendors to deliver it [7,8]. While there have been several prior studies of food safety in Nigeria, a recent review [9] noted that these primarily focused on prepared ready-to-eat foods and only on vendors, with fewer studies examining consumer views or both consumer and vendor views jointly, even though consumer demand has been a major driver of safer food in middle- and high-income countries [10,11,12]. Most studies focused on knowledge or practices, with limited work on beliefs. This is an important gap, as understanding individuals’ motivations and beliefs can be essential in communicating risk and designing effective strategies to change behavior [13,14,15,16,17]. Prior studies have also generally relied on quantitative surveys using closed-ended questions, with limited use of qualitative techniques from anthropology, ethnography, and sociology, which are well-suited to probing in-depth for the “whys” hidden behind actions. Indeed, ethnographic and in-depth qualitative research on food safety is rare worldwide with some exceptions (e.g., [18,19,20,21,22,23] on the related topic of food waste).

To address these gaps, this paper examines the beliefs of consumers and vendors in three traditional markets in Birnin Kebbi, a mid-sized Nigerian city. The focus is placed on traditional markets due to their importance to consumers in LMICs [24] and the challenges they face in controlling foodborne pathogens due to inadequate infrastructure [25], hygiene and storage conditions [26,27,28], and limited oversight [29]. Three specific research questions are considered:

1. What are market vendors’ and consumers’ perspectives on which foods are safe, which are not, and why?
2. For consumers, what is the place of foodborne illness among other health concerns and how big of a role does food safety play among other motivators of food choice?
3. For vendors, how salient is food safety as a concern?

Answering these questions will shed light on the basic drivers of vendor and consumer behavior: their beliefs about what food is safe and how much it matters to them.

## 2. Materials and Methods

This study used an adapted Focused Ethnographic Study (FES) approach. FES has been applied to various public health topics but not yet food safety [30,31]. FES adopts techniques from ethnography, such as in-depth interviewing and cognitive mapping, but applies them in a focused manner, examining specific research questions of policy relevance. It seeks to depict a picture of the behaviors, beliefs, and physical and social environmental factors that shape the topic of interest; in this case, food safety decisions in traditional markets. The research subjects help steer the focus of the research through their responses, which lead to different follow-up questions as well as different potential focuses for the second phase of the two-phase study. The initial phase aims to discover the key themes related to the phenomenon in question, and the second phase confirms and delves more deeply into these. Between the two phases, Phase 1 data are analyzed, and the research questions are refined through iterative reflection and discussion.

The study focused on traditional market vendors and consumers in three markets in Birnin Kebbi, the capital of Kebbi State in northwestern Nigeria. While much of the focus was on general food safety perceptions, some questions asked about specific “focus foods”: rice, maize, cowpea, soybean, fish, green leafy vegetables (GLV, including spinach, moringa, pumpkin leaves, and cabbage; in Kebbi, most GLV are cooked before eating), and beef. The geographic and food focuses were determined based on donor and government priorities and alignment with existing policies and programs. For the choice of the foods, nutritional value, widespread consumption, and covering a diverse range of foods were also important criteria.

Fieldwork was done using trained local interviewers (four teams of two), all of whom were local to Birnin Kebbi and had experience with in-depth interviewing. It consisted primarily of in-person (face-to-face) interviews with consumers and vendors, in two phases. Consumers were defined as those who shop in at least one target market, at least once a month and have primary or shared responsibility for purchasing food for their household. Vendors were defined as those selling at least one focus food in a target market at least once a month. Both consumers and vendors were recruited non-intentionally through market visits. In Phase 1, respondents were recruited only from the city’s main market. In Phase 2, respondents were recruited from all three target markets. The sample size for Phase 1 was 13 vendors and 16 consumers. The sample size for Phase 2 was 24 vendors and 31 consumers. After being selected, potential respondents were asked a set of screening criteria to determine eligibility and willingness to participate, then selected for interviews in line with a set of quotas to ensure the inclusion of a diverse range of respondents. For consumers, quotas were set for men, women, those under age 30, and those over age 30. For vendors, Phase 1 sampling included sellers who sell each focus food, with three vendors each for the foods thought to have the highest food safety risk (beef, fish, GLV) and the others (soybean, maize, rice, cowpea) having one vendor interviewee each. In Phase 2, the focus was narrowed to GLV and fish, chosen due to the potential for high food safety risk, and 12 vendors of each were interviewed. It was intended to interview both female and male vendors; however, no female vendors were available for most of the commodities.

The interview guides and protocol used here were heavily adapted from the original FES manual [30] that focused on young child nutrition. In Phase 1, consumer interviews covered food sources; factors guiding the market, vendor, and food choice; general perceptions of food safety risk; specific food safety perceptions for the seven focus foods; and sources of food safety information. In Phase 2, consumer interviews focused on the perceived risk of illness (including but not limited to food), the process of selecting a vendor, shopping perceptions in general, and specific details on the safety of fish and GLV. Phase 1 vendor interviews examined vendors’ perceptions of their own roles, their relationships with market governance, their perceptions of consumer choices, gender roles, general perceptions of food safety risk, and specific food safety perceptions for the seven focus foods. In Phase 2, they focused on vendors’ motivations for selling food, how food safety related to collaboration and competition among vendors, customer relations, and on specific cues vendors used to address quality and safety for fish and GLV. Not all topics are covered here; some are explored in a companion paper [32]. All interviews also covered sociodemographic characteristics. Interviews included cognitive mapping techniques: free listing (in both phases) and ranking (in Phase 2). In *free listing,* respondents were asked to name all the items in a defined domain; in *ranking*, respondents were asked to rank items on a scale. For certain interview questions, images of the focus foods were used to help respondents picture the food being discussed and ensure uniformity across respondents. Interviews generally lasted 90–120 min.

Data were collected in February–May 2021, following a specific protocol. All interviews were conducted in Hausa, audio-recorded, transcribed verbatim, and translated into English. For structured data (e.g., the free-listing data), these were entered onto paper forms and later transferred to electronic versions. All data were treated with confidentiality, and all participants provided written informed consent. As the research took place during the COVID-19 pandemic, methods were adjusted to protect the safety of the research staff and participants.

Demographic data and the responses to other simple closed questions were tabulated in Excel and/or analyzed in Stata SE15. Interview transcripts were subjected to thematic analysis either through hand-coding or using the software ATLAS.ti. Free-list data analysis used the approach described by [33], using Visual Anthropac 4.9 software (Analytic Technologies). This entailed examining the frequency of an item’s mention and its rank within each list, combined into a joint measure of salience (calculated as the sum of the item’s percentile ranks divided by the total number of lists and ranging from 0 (low salience) to 1 (high salience)). Very similar items (e.g., pasta, macaroni) were consolidated before analysis. Items mentioned often and first or near-first are considered the most salient in the minds of participants. The analysis is presented in the text by synthesizing results across respondents as well as selecting quotations to illustrate either commonly held perspectives or interesting deviations from these. All quotations are presented verbatim from the transcript, aside from small corrections to typographic errors and punctuation, and are accompanied by a short description of the respondent and an anonymous code.

## 3. Results

After discussing the demographic characteristics of the sample, we consider the following key areas of the results: how consumers and vendors conceptualize “safe” and “unsafe” foods, the relevance of food safety risk in driving consumers’ choices, and the salience of food safety as a concern among vendors.

Demographic information for the interviewed consumers and vendors is given in Table 1 and Table 2, respectively. Consumers are about half women. Most are of Hausa ethnicity and Muslim. The sample is relatively highly educated and well off, with only 12% of households estimated as being below the 3.10 PPP international poverty line according to the Poverty Probability Index [34]. Most are married, and the average household has 2.7 children. Nearly all male respondents were the principal income earner for their household, whereas only three women were. Interviewed vendors are all men, nearly all Hausa, and all Muslim. Compared to consumers, vendors are more likely to be Hausa and Muslim, less educated, and more likely to be male. Nearly all are their household’s principal income earner, and most have an additional income source, primarily from farming. All vendors owned their stalls, with about half having employees (usually 1–4 per vendor). As determined by the sampling approach, Phase 1 included vendors of seven foods: three for each of fish, beef, and GLV, and one for each of maize, rice, cowpea, and soybean. About a third of vendors sold other foods besides the “focus foods”. In particular, grain/legume vendors tended to sell multiple types of grains/legumes. In Phase 2, only fish and GLV vendors were interviewed (12 of each). Most GLV vendors (75% of them) sold other vegetables, primarily onions, tomatoes, peppers, and okra.

### 3.1. What Is a Safe Food?

#### 3.1.1. Causes and Consequences of Unsafe Food

Overall, most consumers had a moderate understanding of food safety issues. The main causes mentioned for food becoming unsafe to eat were chemicals and insects. Chemicals were cited both as a general, vaguely defined concern and in relation to specific types: pesticides, fertilizers, and preservatives. Insects were named both as landing on uncovered food (transferring dirt or transmitting disease) and infesting food, though the latter was seen as more a cause of poor quality than unsafe food. Several respondents noted spoilage when food was improperly stored, dust or dirt, and poor food handling. With respect to fish and beef, animal disease and medications were both noted as causes of unsafe food. Not all responses were tied to established food safety hazards or mechanisms from a “scientific” perspective. Only two respondents mentioned bacteria; none mentioned fungi, viruses, or protozoa.

*…those that sell leftover meat have some chemicals they apply on it to prevent it [the meat] from spoiling before the next day. I really don’t know the exact name of the chemical, but I heard it is the type being used on dead bodies. So, if they are not able to sell off their meat and it happens they don’t have a fridge to keep it cool, they use such [chemicals] on it. So, you see if one buys and consumes such kind of meat, it will have a negative effect on one’s health.—C1210, a 25-year-old female consumer with secondary education*

*The meat and the fish and vegetables, because of the way they keep it, it will not make me want to buy… Perching of flies on the particular product, it can bring in diseases. Let say flies perch on it, there would definitely be cholera on it. And sicknesses like typhoid and diarrhea, too.—C2201, a 25-year-old male consumer with post-secondary education*

The main types of symptoms named as caused by food were diarrhea, vomiting, stomachache, and upset/bloated stomach. Less commonly mentioned were headache, fever, loss of appetite, rashes, weakness, brain problems, and death. Some respondents mentioned chronic diseases, like diabetes or high blood pressure, but these were not treated in the analysis as “food safety” concerns, per se. The types of symptoms named generally did not differ across food types. Some of the respondents’ answers were clearly based on knowledge or experience of foodborne illness, while others seemed speculative:

*[Unsafe fish] can cause severe fever and weakness of the body. I think these are what eating such unsafe fish may lead to. Some may lead to death, it can cause…is it yellow fever? I’m not sure… maybe other forms of fever and stomach pains.—C1213, a 28-year-old male consumer with post-secondary education*

#### 3.1.2. Unsafe Foods

In the free listing, participants were asked to list foods they could always count on to be safe and those not always safe. The results of the listings of “unsafe” and “safe foods” are shown in Table 3 and Table 4, respectively. The vendors’ views were slightly different from those of consumers. While beef and local rice appeared prominently on both lists, cowpea (the top concern for consumers) was low on the list of vendors’ concerns, and maize (the third-ranked consumer concern) was absent altogether.

The “most unsafe” food in the minds of consumers was cowpea, named by three-quarters of respondents, usually as the first item. Responses to open-ended questions revealed that this was for two interrelated reasons: cowpea might contain “weevils” (likely actually pod borer insects (*Maruca vitrata*), a major cowpea pest in Nigeria) or other insects (named by 14 of 16 Phase 1 consumers), and potentially unsafe chemicals were used to preserve the cowpea from weevils (12 of 16). In Phase 1, chemicals were mentioned 31 times in relation to cowpea (compared to 16 times in relation to GLV and ≤6 in relation to other focus foods). Insects were seen as an issue both at the point of purchase and in the home.

*[Chemicals on cowpea] come from the person that preserves it, maybe from the farmer, because if they’re preserving it, they put this chemical to prevent insects; if it’s consumed by us, the consumers, it becomes harmful to our health, it can cause sickness, even the cholera that I had talked about, it can even cause death if care is not taken.—C2202, a 31-year-old male consumer with post-secondary education*

While vendors agreed that weevils and chemicals were pervasive issues with cowpea, they also noted that new hermetic storage bags could prevent weevil infestation without chemicals, hence causing them less concern.

Beef was the second-most-unsafe food named by consumers and appeared third on the vendors’ lists. This was due to spoilage over time, being unclean, animal diseases, and animal medications/preservatives. Most respondents recognized beef could spoil over time and be negatively affected by dirt and flies. It was also widely noted that cows might be sick when slaughtered and thus unsafe to eat, or that excess medications or preserved meat after slaughter could have negative consequences for consumers.

*We also have dishonest persons that sell dead livestock to people, for instance when a livestock has been hit by a moving truck, they instantly pick up the dead livestock and prepare it for sale to unsuspecting consumers. Then we have livestock that are sick; after several treatments prove abortive, the owner will decide to sell it off to meat sellers. So, you can agree with me that such [things] can cause harm to humans. Secondly, the place they spread the meat before selling, like a glass covering, flies and other insects follow the meat and contaminate the beef meat, and if care is not taken, harmful substances can easily be consumed.—C1211, a 38-year-old male consumer with post-secondary education*

However, some trusted that the existing systems kept beef safe or felt beef was generally safe:

*In every market, when they bring a cow to be slaughtered, they do all the repairs in an open place where everyone can see… When it’s done that way, such beef does not cause harm to people. And also, they don’t allow the meat to stay over till the next day; even when it does, they make sure they roast and sell it off the same day.—C1203, a 50-year-old woman with primary education*

The next six items (maize, local rice, yam, sorghum, GLV, and tomato) were named by 3–4 consumers, seldom among the first items. While there are some who see them as unsafe, they are not of high salience. Local rice and GLV were also highly ranked by vendors as unsafe. For local rice, this concern was driven by the presence of stones. It was the only food for which physical contaminants were commonly mentioned. An important distinction respondents made was between *foreign* and *local* rice: foreign rice was imported, branded, and often packaged, whereas local rice was often bought loose. Several vendors and a few consumers noted that the foreign rice was generally free of foreign bodies like stones, compared to local rice:

*Take foreign rice, for example; when you are eating it, it is safer than our traditional rice that’s filled with stones. When you chew stone in it, you will just feel like not eating again. Any food seller that I buy rice from, and I felt stones in it while eating, I will not buy from her again.—V1109, a 35-year-old male GLV vendor with no formal education*

Indeed, some respondents saw a general connection between foreign food and safety or quality:

*…Foreign rice, it is safe to eat. Foreign canned tomatoes, foreign canned fish, foreign salad cream is safe, foreign Bama is safe, but our own Nigerian made is unsafe…. it is not comparable with Nigerian made, foreign is higher quality. In my own opinion, [the] Nigerian Government does not care about what is produced here, while their own [foreign] government makes sure quality foods are produced for its citizens.… I do not know the reason, but foreign is better than Nigerian made. I am not in that [foreign] country, but I know [its] government makes sure and makes it mandatory to produce quality products, the company wants to maintain good reputation and maintain their customers. But here, since we don’t have a choice, anything produced will sell, consumers will buy.—V1107, a 43-year-old grain/legume vendor with primary education*

For GLV, some consumers expressed no concern; as one man noted, “I have not seen anyone that ate green vegetables and got harmed, and I don’t think they will harm anyone” (C1214, 30 years old). The majority, however (13 of 16 in Phase 1; 18 of 31 in Phase 2), did have concerns related to insects (worms damaging the leaves or being inside, flies landing and spreading disease) or chemicals (fertilizer, pesticide, or herbicide). Some consumers also mentioned washing with unclean water or spoilage. Dirt or sand was noted as a quality issue but not necessarily a safety one.

*If one eats a bad vegetable, the person will fall sick [with] fever.… And also when one eats vegetables that has worms, it will lead to vomiting and stooling, then fever.—C1206, a 30-year-old female consumer with secondary education*

*Yes, there is a problem when one eats those [green leafy vegetables] that were grown with chemicals or have chemicals applied on them…. It can cause harm to the body. It can cause stooling, vomiting and severe fever.—C1213, a 28-year-old male consumer with post-secondary education*

In general, consumers recognized that food safety concerns might arise with GLV in certain circumstances, but did not see them as common, and those concerns did not prevent consumption. Most food safety issues were seen as manageable with in-home behaviors like washing or cooking.

The remaining foods (a mix of grains, oils, snacks, and vegetables) were named by only 1–2 people each. Interestingly, several types of food that experts might describe as a high risk for contamination (fish, chicken, eggs, dairy; other fresh fruit and vegetables) were not named, while several of those named (particularly the grains, yam, and oils) are generally not associated with high risk in scientific risk assessment. Aside from rice, where the potential risk was physical damage (i.e., broken teeth), all “unsafe” foods were associated with similar consequences—gastrointestinal symptoms and, less commonly, fever or headache—regardless of the perceived hazard.

#### 3.1.3. Safe Foods

For “safe foods” (Table 4), grains, roots, tubers, and legumes were commonly named. Several of the most salient “safe foods” were industrially packaged and/or processed: pasta, noodles, imported rice, Semovita, tinned foods, and couscous (Maltina, Semovita, and tea were also named by one vendor each). Several consumers mentioned that either being packaged or “from a company” was a reason for food to be seen as safe, suggesting higher trust in these types of industrially produced products:

*The reason why I said tinned milk [is safe,] it’s because is made from the company, because it has passed through quality control before selling it out.—C1212, a 30-year-old male consumer with post-secondary education*

*Mostly we prefer packaged foods because they’re safe… I prefer packaged foods than open foods because [of] how they are being treated and being preserved. When you get packaged foods, you can actually get it anywhere, it is the same thing. But when you go to a particular customer [i.e., vendor] to get vegetables or fish, they are open foods…. You [must] have trust with someone you will know that, that person [is] clean and neat.—C2201, a 25-year-old male consumer with post-secondary education*

*[Instant noodles are] made by companies, so they wouldn’t sell something of low quality.—C2219, a 46-year-old male consumer with post-secondary education*

However, a few named packaged/industrially processed foods, particularly Semovita (blended flour) as foods of concern due to chemicals or prolonged storage.

Surprisingly, fish, beef, and GLV all also appeared on consumers’ “safe foods” lists. For GLV, this is with slightly less salience than as an “unsafe food”, suggesting divided opinions, whereas for beef the salience is considerably lower. The appearance of cowpea on both lists is also surprising. The respondents’ commentary indicates that this reflects not that cowpea is *always* safe but rather that with the respondent’s care and attention, safe versions can be identified. Similarly, vendors made a separation between the risk associated with certain foods in general and risk they attached to the same foods *when sold by them*, offering explanations like “I only sell the good ones”.

The different types of hazards associated with each of the seven focus foods are shown in Table 5, which indicates the number of times that a hazard was mentioned in connection with each food in the Phase 1 consumer interviews. This shows some variation across food types (discussed in the next section for specific foods) but also certain general patterns: a high level of concern related to chemicals and insects and no mention of other types of contaminants (e.g., bacteria).

### 3.2. What Is the Place of Foodborne Illness Concern among Consumers’ Choice Drivers?

#### 3.2.1. Consumers’ Health Worries

When asked about a general concern for their family’s health, nearly all respondents noted concern about certain health issues. Chiefly, these included malaria and diabetes, followed by COVID-19 and heart disease/high blood pressure. About 15 of 31 Phase 2 consumers mentioned foodborne disease without prompting. This was primarily typhoid, cholera, or general (unspecified) illness caused by food. About half connected typhoid or other causes of diarrhea to unclean/spoiled food. Connections to unclean water or environment were equally common. When asked specifically which diseases caused diarrhea and/or vomiting, most respondents were quick to note typhoid, cholera, and malaria, but some also mentioned “fevers” (a general term for illness). In free listing for this topic, malaria was the most commonly named cause of diarrhea and/or vomiting, named by 74% of respondents, commonly as a first response; typhoid, stomachache, and cholera were named by 40–50% of respondents, while 35% noted “fever” and three noted ulcers. Typhoid and cholera seemed to be used as general terms for gastrointestinal illnesses as opposed to those caused by specific bacteria (e.g., *Salmonella typhi, Vibrio cholerae*); a similar vagueness in definition may also be the case for malaria [35]. Notably, while chemicals were commonly mentioned as hazards, no health concerns related to their impacts, such as acute or chronic toxicity or cancer, were mentioned. Foodborne illness is thus widely recognized but not among the top health concerns for most consumers, and they confuse it with other types of illness, with causes not clearly attributable to food.

#### 3.2.2. Level of Concern around Food Safety

When asked directly if they ever worried that foods could be harmful to eat, about half of Phase 1 consumers replied yes, though four of those expressed only mild concern. Others remarked that they had never thought of it, never seen such a thing, or did not believe it could happen. Some of those who expressed no concern reported that this was because they felt they were already making the appropriate choices to avoid unsafe food. In contrast, when asked directly in Phase 2 whether foods could cause diarrhea and vomiting, most respondents agreed and could list foods they associated with these symptoms.

Only about one-third of Phase 1 respondents reported ever having personally experienced foodborne illness themselves or in family members. Those who mentioned one incident were likely to mention several—no respondent mentioned it happening only once—and were slightly more likely to be concerned about food safety as an issue.

*I have never encountered any problem since I started eating vegetables, and no one has told me if they had any harm [from] eating it. And for rice and even fish, no problem, and besides, there is medication when one falls sick.—C1203, a 50-year-old female consumer with primary education*

*[Once, a man] bought meat that has been in a nylon [plastic bag] for a very long time. When he brought it home, my family was given part of the meat, and his own family ate, too.… They were all taken to the hospital as a result of eating the meat. Everyone that ate that meat felt sick.—C1205, a 53-year-old male consumer with no formal education*

*In my hometown… It happens that some people consumed particular beans [cowpea], and it led to the death of a number of persons. But immediately it was discovered, action was taken before it became worse…. I think what led to it was the chemical that was added to the beans, it expired when it was not supposed to, and it was sold out. So, the woman that bought the beans unknowingly cooked them for consumption.—C1210, a 25-year-old female consumer with secondary education*

#### 3.2.3. The Role of Food Safety in Consumers’ Reasons for Choosing Food, Markets, and Vendors

Consumers’ main reasons for choosing to eat particular foods included traditions and habits, family preferences, variety, taste, and affordability/price constraints (particularly for meat or fish). When asked about particular foods that were important for feeding the family, respondents mentioned those that were sources of energy, protein, and vitamins. When asked about the general reasons or processes for choosing food, without referring to safety, no consumers mentioned safety or cleanliness as a factor.

When it came to choosing a market, price was the primary motivator, followed by product availability and a convenient location. Familiarity/habit and necessity were also mentioned. High prices were mentioned as the most common cause of an unsatisfying or unsuccessful market trip. Only two of 16 Phase 1 consumers, both women, brought up issues related to food safety or cleanliness unprompted when it came to market choice, citing it as a positive attribute that markets were “neat” or “clean”.

The choice of a vendor was also driven largely by price (13 of 16 Phase 1 consumers), including discounts or free goods. Credit provision was mentioned by one quarter of respondents. While not a key concern for all shoppers, credit was an important motivator for some, particularly in a context of often increasing and unpredictable prices, and could lead to ongoing vendor-shopper relationships. Beyond price, the main characteristics motivating the choice of a vendor related to interpersonal qualities: niceness, politeness, good customer relations, and patience (which consumers described as related to a vendor’s willingness to bargain and discuss). Four (of 16) Phase 1 consumer interviewees brought up vendor cleanliness unprompted when asked about general criteria relating to vendor choice. This was usually presented as one criterion among several, but a necessary one:

***The first thing I look out for is neatness**, then I also look at a vendor that has all that I need the moment I go to him to get my items. Another thing I look out for in a vendor, is a vendor with good sense of humor, with good human relations. I also look at how long the vendor has been into the business. These are the main qualities I take note of.—C1211, a 38-year-old male consumer with post-secondary education [emphasis added]*

*I check the quality of what I want to buy and then the price…. I will visit 2 to 3 shops and then go for the cheaper one. For me, though, I go for quality before I make a purchase, and I look **for how neat it is and the surroundings** for the entire products I am going for, that is what I normally do.—C1215, a 37-year-old male consumer with post-secondary education [emphasis added]*

When the topic of foodborne illness was broached more explicitly in Phase 2, respondents were much more likely to mention cleanliness as something they sought in a market or vendor (26 of 31 respondents). Moreover, when asked to rank five potential ways that a vendor could attract customers, cleanliness was commonly rated first (above low prices, patience, tasty food, and offering credit).

### 3.3. How Salient Is Food Safety as a Concern among Vendors?

To gauge the importance of food safety to vendors, we asked them to list the qualities they believed were required for success in vending food. “Cleanliness” and “having a washed/clean product” were the only responses with a direct bearing on food safety that the listing exercise produced, each named by less than 10% of vendors and appearing at a low position on the list, suggesting low salience. Far more salient are aspects of vendor character (e.g., patience, honesty, friendliness) and competitive pricing. In Phase 2, vendors were asked to rank cleanliness (of the shop and/or their person, and used as a proxy for safety, which vendors tended to intermix with quality) as a way to attract and keep customers, as compared to other qualities that were noted in Phase 1 as being important aspects of being “a good vendor”. The results are shown in Table 6. Patience is emphatically at the top of the list, followed by lower prices, with cleanliness in the middle.

Interviews also explored the topic of food safety through exploratory, open-ended questions about vendors’ own food safety concerns and the concerns expressed to them by shoppers. Five of 13 vendors stated that the safety of their own foods for sale was not an issue that concerned them. Among those who acknowledged a concern, the issues identified were familiar from the consumer interviews: chemicals (fish, GLV), stones and dirt (soybean), and animal health (beef). In general, vendors intermixed food safety with food quality, which they construed in general terms as “freshness”. While freshness may be associated with food safety (e.g., through reduced risk of damage, spoilage, or exposure to conditions unfavorable to food safety), safety-enhancement was not the main benefit associated with freshness by vendors. Instead, a fresh product was perceived to be easily recognized and appreciated by shoppers, making it an effective way to maximize sales. This was particularly important for GLV, for which apparent freshness was seen as an important motivator for consumers. Some vendors gave freshness primacy, even above price, reasoning that fresher food permits vendors to start the price conversation with shoppers on a different footing:

*One of the ways to be noticed and selected by customers is that–imagine that this is my table and there are very fresh vegetables laid out on the table. Naturally, the freshness of the vegetable is one of the things that affects customers. Even before they ask the price, the goods are what will first of all captivate them… One of the things a shopper expects from a trusted vendor is good quality product. Then, after that, we can talk about good price.—V2113, 35-year-old GLV vendor with primary education*

Nine of 13 vendors stated that shoppers sometimes raise food safety issues, but their accounts of these encounters make it clear that much of this consumer concern is directed not at food safety strictly speaking, but rather food quality. Averaging the estimates made by vendors, less than a third of customers were said to ask questions about either the quality or safety of their food. Women were cited by some vendors as being more likely to have concerns or be more discerning, as were urban salaried and educated shoppers, whereas the poorer consumers were noted as less concerned. Regarding specific foods, vendors did note that safety-related issues shaped customers’ choices for certain foods, primarily cowpea (chemical and weevil free) and rice (free of stones). For beef, two vendors noted the importance of a clean sale environment and a clean vendor in communicating safety/quality and encouraging customers to purchase, which was not the case for any other focus foods. As one beef vendor noted, “Meat with dirt is not healthy … because the business requires neatness. From where we buy up to where we sell, needs to be clean” (V1112, age 25, secondary education). However, some vendors (particularly of fish and maize) saw safety as an unimportant issue for shoppers.

In sum, while vendors were aware of food safety in a general sense, it appeared to be an issue of, at most, moderate concern.

## 4. Discussion

This study has examined the food safety beliefs of vendors and consumers in a mid-sized Nigerian city, painting a rich, in-depth picture and offering insights for the design of interventions. Regarding the first research question, on vendors’ and consumers’ perceptions of what causes unsafe food and which foods are safe and unsafe, a fairly clear picture emerges. The main perceived causes of unsafe food, at least among consumers, were chemicals (including fertilizers, pesticides, herbicides, and preservatives) and insects. Less commonly named were spoilage, dirt, and poor handling. Few respondents mentioned bacterial contamination or explained in detail the mechanisms through which it could occur. While bacterial illnesses such as typhoid were widely mentioned as a cause of diarrhea and vomiting, these were not necessarily linked to food in consumers’ minds. There was also some conflating of foodborne illness with other food-related aspects of health (e.g., high blood pressure). Respondents agreed strongly that certain foods were less likely to be safe than others, with cowpea (due to chemical contamination and insects), beef (due to chemicals and spoilage), GLV (due to chemical contamination and insects), and local rice (due to stones) being commonly named. The main consequences of unsafe food mentioned were gastrointestinal symptoms and, less commonly, fever or headache. The chief food safety concern among consumers was clearly chemical contamination or weevil infestation of cowpea, though this was not a main concern among vendors.

Contamination of cowpea in Nigeria with organophosphate pesticides is a documented issue, potentially causing health issues and leading the European Union to suspend dried bean imports from Nigeria since 2013 (and garnering local media attention as a result) [36,37,38]. However, the relatively high level of concern over this hazard among consumers is not warranted according to the WHO analyses of the burden of foodborne illness, nor is the concern related to chemical contamination of GLV and beef. Instead, diarrheal diseases are by far the most important contributor to the burden of foodborne disease in the region, followed by helminths and invasive bacteria. Chemicals and toxins are responsible for only about 2% of the burden [39]. The main foods flagged by experts as food safety concerns in Nigeria are thus mostly animal-sourced foods, particularly pork (due to pig tapeworm, *Taenia solium*), poultry, and beef, as well as fruit and vegetables, due primarily to bacterial contamination (e.g., with *Salmonella* and pathogenic *E. coli*) [6]. The prioritization of chemical hazards over biological hazards is not uncommon: similar concerns have been found among consumers in many LMICs [40], such as Kenya, Vietnam, and Ghana [41,42,43,44,45,46], as well as in high-income countries [47]. This may be due to the human tendency to overweigh risks that are poorly understood or beyond one’s perceived personal control, such as chemical contamination [48]. Indeed, Grace and colleagues argue that non-experts systematically overestimate the impacts of chemical hazards and underestimate those of biological ones [6], and wide gaps between expert and lay person perceptions on food safety have been widely documented [49,50]. Be this as it may, food safety interventions aiming to steer consumers’ and vendors’ choices will likely need to accommodate these existing views in terms of what hazards they focus on when framing the issue.

An additional finding of note under the first research question was the prominence of processed and packaged foods among the foods listed as “safe”, as well as the tendency for some respondents to equate “foreign” or “industrial” foods with being safer than “local” or unprocessed foods. Similar findings for packaged foods have been identified in other LMICs [40]. The association of local food with “less safe” has also been noted in Brazil [47], while researchers in high-income countries, as well as Vietnam, have found the opposite association (local foods being seen as intrinsically safer [41,47,51]). The results also align with the conclusions of a recent review of ethnographic research on food safety that noted that consumers tend to use binary categorizations (e.g., at home versus outside foods) to think about food and food safety [52]. While they may indeed be at less risk of contamination (especially from bacteria, viruses, and helminths), many of these “safe” foods (e.g., instant noodles, pasta, couscous) are relatively highly processed and nutrient-poor when compared to the “less safe” foods (e.g., beef, GLV, cowpea). Indeed, the prioritization of processed, packaged food was hypothesized as one pathway through which food safety concerns could have a negative influence on nutrition [53].

Turning to the second research question, the results give a somewhat ambiguous answer regarding how important food safety is as a concern and motivator of choice among consumers. The study found a fairly low level of salience compared to other concerns *when the topic was not specifically primed or prompted*. However, once consumers were asked directly or had been primed on the topic, it was much more prominent, with the majority of consumer respondents opining on the importance of vendor and market cleanliness and naming strategies to avoid unsafe food. In general, however, consumers’ concerns about food safety did not seem to be overriding, i.e., they recognized that food safety concerns might arise with certain foods in certain circumstances, but not commonly, and those concerns did not prevent consumption of that food. Most foods were seen as safe, and it was rare that consumers reported avoiding consumption of a specific food due to safety or quality issues. Moreover, while unsafe food was associated with gastrointestinal diseases, it was not seen as the *only* cause of any gastrointestinal symptoms, which people also associated with malaria and unclean water. Respondents had rather vague and flexible ideas about types and causes of illnesses, including foodborne ones, and surprisingly few reported direct experience of getting sick from food.

Jointly, these results align to findings from lower-income countries that food safety is rarely a dominant concern of either the public or policymakers [54]. From an intervention perspective, they suggest that, while food safety is unlikely to be a top-of-mind key motivator of consumer choice at the moment, there is some awareness and recognition of its importance, and people can likely be convinced to make it more central in decision making if given the right cues and motivators. However, in so doing, any intervention will need to be cautious in linking safer food with less illness. Given consumers’ partial understanding of gastrointestinal illnesses’ causes and inability to clearly separate foodborne illness from other illnesses, they might not naturally be making a connection between food and disease, and a reduction in general gastrointestinal illness could be attributed by them to other causes, such as malaria prevention. Conversely, program-related reductions in the transmission of foodborne pathogens could remain obscured in a setting where illness persists, e.g., due to sanitation conditions.

In addition, food safety will always be competing in consumers’ minds against other salient motivators of food and vendor choice [41]. In particular, it was clear that price is a strong motivator for consumers, central to driving vendor and market choice. Given this primacy, it seems unlikely that many consumers will be willing to set aside price concerns in order to prioritize safety. This is particularly true for women. Other data from the interviews (not discussed in depth here) indicated that women have less agency when it comes to choosing to pay a higher price (as they are often using their husband’s money), bargain harder, and particularly appreciate having change left over after shopping. Moreover, credit also proved to be a key motivator for some consumers, implying less flexibility in their budget allocations. These findings align with research that has found food safety to be a limited motivator of consumer choice in other LMICs [55,56,57]. Research elsewhere in Nigeria has found some willingness to pay for safer food but not necessarily among lower-income consumers (e.g., [58]).

Regarding the final research question, salience of food safety among vendors, we found that most vendors did not see food safety, cleanliness, or hygiene as a key trait of a successful vendor and that just over half of vendors had any concern about the safety of their food. Similarly low prioritization of food safety, despite documented risks, has been found among meat sellers in Tanzania [59]. Vendors’ perceptions of food hazards were largely similar to those of consumers, with the exception of cowpea, a product for which vendors acknowledge consumers’ concerns but (at least some) feel that they have adequate technology to mitigate through the use of improved storage bags [60].

Vendors also did not consider cleanliness to be a top way to attract customers and estimated that only about one-third of customers asked questions about either quality or safety. Whether this directly reflects that only one-third of customers are concerned about quality or safety is not clear. Customers may still have evaluated it through visual inspection, they may have been skeptical of whether vendors would be knowledgeable or honest about food safety (especially given the class difference between the two groups), or they may have prioritized bartering on price over discussing quality. Indeed, research in Ghana has noted that customers are hesitant to voice food safety concerns to vendors [56]. For most vendors, the concepts of safety and quality were intermixed, related to the concept of “freshness”, which was visually observable and seen as an important driver of choice. This result is similar to the abovementioned Ghana study, which found that vendors and consumers focus on “appearances” as opposed to actual safety determinants [56], and work in Vietnam that has found that consumers prioritized perceived freshness of vegetables (and convenience factors) over markets with modern food safety infrastructure [61]. Indeed, both consumers’ and vendors’ conceptualizations of “food safety” were overlapping with other aspects of quality, such as insect damage, and few consumers or vendors articulated a clear definition of “safety” that was separate from these issues and directly and uniquely linked to foodborne disease. Food safety can be seen as one aspect of food quality: high-quality food must be safe (though safe food does not necessarily imply high-quality food). However, consumers and vendors in this study did not make a clear distinction between the two and instead tended to speak of quality and safety concerns somewhat interchangeably, or as occurring along a continuum. This aligns to findings from other LMICs [62] and is important to consider when framing behavior change interventions.

This study has some limitations. First, the sample was small and not fully representative of the local population. To increase reliability and validity of our data, we used a variety of ethnographic techniques, question types and question ordering, and prompted about specific foods; however, the food safety topic may be affected by memory or personal experiences or perceived personal control over food safety, so the ambiguity of some of our findings may be due to inter-respondent variability. All vendors interviewed were male due to an inability to find eligible female vendors to interview. In addition, the consumer sample was likely somewhat more affluent than the total Birnin Kebbi population. Nigeria’s urban poverty rate is estimated at about 18%, whereas about 12% percent of this sample (using a somewhat more generous threshold) were found to be poor. Similarly, the sample is also somewhat more educated than the average for urban Nigeria, according to the 2018 Demographic and Health Survey [63]. The population studied is thus more representative of the middle class (as opposed to lower-income consumers) and may have a greater ability to access information related to food safety and be able to be discerning when shopping (due to greater purchasing power). These differences should be taken into consideration when interpreting the results, and a large, representative-sample survey could help to contextualize them better. Moreover, we did not examine home food preparation practices; our results are primarily about the market context. Finally, the focus on vendors of specific commodities may have produced biased results: while these vendors covered many major food groups (legumes, grains, vegetables, meat, and fish), they omitted dairy, egg, and fruits as well as ready-to-eat foods.

## 5. Conclusions

We conclude by drawing on these insights to suggest certain recommendations for improving food safety in Birnin Kebbi and similar settings. First, the results suggest that there is some awareness of food safety and interest in avoiding foodborne disease. Interventions, such as public health campaigns, can seek to make this a more salient motivator by focusing on the potential risks of consuming unsafe food (or the potential benefits of avoiding it), perhaps in relation to economic costs and benefits, which appear to be a strong motivator for consumers and vendors alike. Second, education campaigns for both consumers and vendors can work to disambiguate actual “safety” aspects, and cues to identify hazards and risks, from more general “quality” aspects, as the two concepts are currently intermixed in many people’s minds. Finally, there is a critical role for government interventions at the market level that can improve food safety infrastructure. Vendors are likely unable to engage in such upgrading alone, given the costs, and it is important that it is done with minimal impact on consumer prices in order to ensure such changes are not biased against the poorest and most vulnerable.

In summary, this study has offered an in-depth perspective on how consumers and vendors in Birnin Kebbi, a mid-sized Nigerian city, understand and prioritize food safety within their everyday decisions, especially at point of purchase. In short, it found that food safety was conceived primarily in terms of chemical hazards, associated with certain foods and food traits. Food safety overlapped with issues of food quality and freshness. While both consumers and vendors had some awareness and concern about food safety and foodborne illnesses, they were not top priorities for either group and tended to be intermixed with aspects of food quality or other types of illnesses. As Nigeria continues to modernize and urbanize, with food value chains lengthening and increasing consumption of ready-to-eat foods, food safety risks are likely to become only more acute, so it will be crucial to raise the saliency of food safety among vendors and consumers. To address this challenge, understanding vendors’ and consumers’ current views on food safety, and designing behavior change approaches to accommodate and build upon them, will be crucial.

## Figures and Tables

**Table 1:** Consumer demographic characteristics (*n* = 47).

Respondent Characteristics	
Gender	Male (49%), female (51%)
Average age (range)	33.7 (22–64)
Ethnicity	Hausa (47%), Zuru (30%), Fulani (15%), Igbo (6%), Other (9%)
Religion	Muslim (62%), Christian (38%)
Highest education completed *	Primary (94%), Tertiary (53%)
Marital status	Married (monogamous)—66%; married (polygamous)—6%; single—26%, widowed—2%
Principal household income earner	45%
Occupation	Professional/Managerial—30%; Small business owner/entrepreneur—15%; Not employed outside home—23%; Sales/services employee—11%; Petty trader, hawker—6%; unskilled labor—2%, technical labor—9%, agriculture—4%
**Household Characteristics**	
Avg. household size (range)	6.2 (1–19)
Avg. number of children (range)	2.6 (0–11)
Home has electricity	91%
Pct. poor (1.90 PPP)	2%
Pct. poor (3.10 PPP)	12%
Household owns car	32%
Household owns mobile phone	98%
Household has improved toilet	91%
Farms or owns farmland	55%

* Note: under Nigeria’s education system, primary and secondary school each consist of six years.

**Table 2:** Vendor demographic characteristics (*n* = 37).

Vendor Characteristics	
Percent male	100%
Average age (range)	40 (22–65)
Ethnicity	Hausa (95%), Fulani (5%)
Religion	Muslim (100%)
Pct. completing primary school	51%
Pct. completing secondary school	22%
Pct. completing tertiary school	3%
Avg. years vending (range)	19.2 (5–43)
Respondent is household’s principal income earner	95%
Respondent has another income source	70%
Other income sources	Farming or livestock (23); selling other food/goods (2); contractor (1)

**Table 3:** Free-list results for “unsafe foods”.

Food	Percentage of Respondents Mentioning	Average Rank	Salience
Consumers (*n* = 16)			
Cowpea	75.0%	1.33	0.667
Beef	50.0%	3.75	0.233
Maize	31.3%	2.40	0.22
Local rice	31.3%	3.20	0.162
Yam	31.3%	3.20	0.161
Sorghum	25.0%	3.25	0.126
GLV	25.0%	4.50	0.116
Tomato	25.0%	4.75	0.103
Groundnut Oil	12.5%	3.00	0.083
Cabbage	12.5%	2.50	0.078
Palm Oil	12.5%	2.50	0.086
Kanzo (scorched rice)	12.5%	2.50	0.078
**Vendors (*n* = 13)**			
GLV	76.9%	3.10	0.506
Local rice	69.2%	3.00	0.475
Beef	61.5%	2.88	0.431
Fish	53.8%	2.86	0.366
Tomato	38.5%	3.00	0.293
Fura *	23.1%	4.67	0.117
Fruits	23.1%	5.33	0.066
Millet	15.4%	5.00	0.078
Cowpea	15.4%	1.00	0.154

Note: items named only once are omitted. Of the study focus foods, this included soybean and dried fish. “GLV” includes specific types of GLV as well as “leafy greens” or similar and “vegetables” not otherwise specified. Those foods in bold also appeared in Phase 2 consumers’ top ten foods named as causing diarrhea or vomiting. * Fura are millet dough balls, often used in a milk-based drink fura da nono. As the samples are fairly small, results should be interpreted with caution.

**Table 4:** Free-list results for “safe foods”.

Food	Frequency	Average Rank	Salience
Consumers (*n* = 16)			
Imported Rice *	93.8%	2.13	0.783
Cowpea	43.8%	4.14	0.226
Spaghetti/Macaroni	43.8%	2.86	0.306
Yam	43.8%	4.14	0.247
Eggs	31.3%	3.80	0.205
Maize	31.3%	3.40	0.204
Fish	25.0%	6.00	0.081
Beef	18.8%	4.33	0.121
Millet	18.8%	4.67	0.079
Indomie (instant noodles)	18.8%	3.33	0.122
GLV	18.8%	6.67	0.065
Sweet potato	18.8%	5.33	0.063
Red pepper	18.8%	5.00	0.092
Tomato	18.8%	6.00	0.061
Irish potato	18.8%	5.67	0.049
Semovita (semolina flour)	18.8%	2.33	0.123
Onion	12.5%	3.50	0.083
Soybean	12.5%	3.50	0.036
Groundnut oil	12.5%	1.50	0.117
Dried fish	12.5%	5.00	0.06
Couscous	12.5%	4.00	0.058
**Vendors (*n* = 13)**			
Imported Rice *	61.5%	2.00	0.477
Instant noodles	30.8%	2.75	0.213
Millet	30.8%	3.25	0.192
Maize	30.8%	1.50	0.269
Spaghetti/Macaroni	23.1%	4.00	0.099
Tinned fish	15.4%	3.50	0.065
GLV	15.4%	3.00	0.081
Cowpea	15.4%	2.50	0.096
Groundnuts	15.4%	3.00	0.11
Tinned Tomatoes	15.4%	2.00	0.126
Fish	15.4%	3.00	0.069
Sorghum	15.4%	4.00	0.038
Wheat	15.4%	5.00	0.06

* This includes those who named “safe rice” more generally than imported rice. While many respondents made it explicit that the safe rice was the imported rice, some referred instead to ‘the one without stones’; this was coded as imported rice based on the responses to open-ended questions. As the samples are fairly small, results should be interpreted with caution.

**Table 5:** Main hazards cited by consumers by type of food.

	Cowpea	Maize	Rice	Soybean	Beef	Fish	GLV
Animal diseases	NA	NA	NA	NA	12	2	NA
Animal medications	NA	NA	NA	NA	5	3	NA
Chemicals	31	5	5	0	4	6	16
Insects	20	4	1	5	3	2	8
Physical contaminants	2	1	8	3	1	2	1

Note: Number of times mentioned, across all Phase 1 consumer interviewees; NA = not applicable.

**Table 6:** Results of vendor ranking exercise for “ways to attract and keep customers” (*n* = 24).

Trait	Average Rating (1 = Lowest, 5 = Most Important)
Being more patient	4.33
Maintaining lower prices	3.13
Maintaining a cleaner stall	3.00
Being more trustworthy	2.38
Allowing purchase on credit	2.04
